# Atypical Presentations of Huntington Disease‐like 2 in South African Individuals

**DOI:** 10.1002/mdc3.14052

**Published:** 2024-05-09

**Authors:** Heena Narotam‐Jeena, Mark Guttman, Ludo van Hillegondsberg, Riaan van Coller, Amanda Krause, Jonathan Carr

**Affiliations:** ^1^ Division of Neurology, Department of Medicine University of Stellenbosch Cape Town South Africa; ^2^ Centre for Movement Disorders, Markham Ontario, Division of Neurology University of Toronto Toronto Ontario Canada; ^3^ Department of Neurology University of Pretoria Pretoria South Africa; ^4^ Division of Human Genetics, National Health Laboratory Service and School of Pathology, Faculty of Health Sciences University of Witwatersrand Johannesburg South Africa

**Keywords:** chorea, HDL2, Huntington

## Abstract

**Background:**

Huntington disease‐like 2 (HDL2) is a neurodegenerative disorder, affecting only individuals of African ancestry. Full penetrance occurs in individuals with 40 repeats or more.

**Objective:**

To describe the phenotypic variability of HDL2 in a group of mixed ancestry individuals from South Africa.

**Methods:**

Eight patients were assessed with analysis of repeat size and magnetic resonance brain imaging. We applied the Unified Huntington's Disease Rating Scale (UHDRS), but in deceased patients (4), this was estimated from video material.

**Results:**

Cognitive domains were more severely affected than motor; UHDRS motor scores were notable for bradykinesia, and to a slightly lesser extent, for rigidity and dystonia; a single patient had marked chorea. Repeat lengths ranged from 45 to 63 (median, 52).

**Conclusion:**

This South African group of mixed ancestry HDL2 individuals presented with severe cognitive and behavioral impairments, with lesser degrees or absence of chorea. This presentation is possibly related to large repeat sizes.

Huntington disease‐like 2 (HDL2) is an autosomal dominant neurodegenerative disorder, with progressive movement and cognitive dysfunction and has been reported to be clinically indistinguishable from Huntington's disease (HD).^1^, ^2^ Involuntary movements, especially chorea, are typically present, associated with progressive dementia and with rigidity and bradykinesia predominating in later stages of the disease.^3^ Unaffected individuals have 6 to 28 CTG triplets, whereas full penetrance occurs in individuals with 40 repeats or more.^4^ HDL2 appears to affect only those with African ancestry from sub‐Saharan Africa, although cases have been identified in the Americas and Europe.

In the Western Cape province of South Africa, individuals of mixed ancestry are also affected, their ancestry being derived from African populations (San, Khoi‐Khoi or Bantu‐speaking), European immigrants, and from Madagascar, the Malaysian archipelago and India.^5^

This study highlights unusual phenotypic features of HDL2 in the mixed ancestry population of the Western Cape, a group with large repeat sizes, and prominent cognitive deficits, with less pronounced chorea.

## Methods

This case series was conducted at a large state hospital in Cape Town between January 2006 and September 2021. Eight adults with genetically confirmed HDL2 were included; retrospective data were collected for half of the study participants, as they were deceased. The Unified Huntington's Disease Rating Scale (UHDRS) and the Montreal Cognitive Assessment (MoCA) tool were used for clinical assessment. In two deceased patients, Mini‐Mental State Exam (MMSE) scores were used (noting that the MMSE and MOCA lack normal controls in this population). All participants with genetically confirmed HDL2 (ie, CTG repeat lengths of 40 or more) had their DNA analyzed using polymerase chain reaction and capillary electrophoresis, and GeneMapper Software 5 (Applied Biosystems, Foster City, CA) was used for fragment analysis and sizing.

Genetic counseling was provided by trained genetic counselors to all participants. The UHDRS (Table 1) was carried out by two movement disorder experts (J.C., R.v.C.), and where a numerical difference of ≥2 between raters was identified, video material was further reviewed by a rater with specific experience in the UHDRS (M.G.) Median repeat values between the patients reported here and those previously published from Gauteng were analyzed with a Mann–Whitney test^2^.

**TABLE 1:** Summary of AAO, repeat numbers, cognitive scores and UHDRS scores (summed ocular scores and scores from four of the motor components of the UHDRS)

Participant	CTG repeat	AAO	MoCA MMSE	Years of Schooling	Ocular	Rigidity	Bradykinesia	Dystonia	Chorea
A‐II‐3	16/59	25	Nd	Unk	Nd	Nd	0	Nd	2
A‐II‐6	14/49	38	14^a^	8	11	Nd	2	9	3
A‐III‐10	14/52	42	13^a^	5	Nd	3	3	4	0
A‐IV‐7	14/63	18	1	11	24	4	12	6	2
A‐IV‐8	14/59	19	10	12	14	2	3	0	1
B‐II‐5	14/49	48	13	6	17	5	9	4	4
B‐III‐5	15/51	38	2	8	16	3	12	4	0
C‐III‐5	23/45	43	23	6	8	4	4	12	9

Abbreviations: AAO, age at onset; UHDRS, Unified Huntington's Disease Rating Scale; MoCA, Montreal Cognitive Assessment; MMSE, Mini‐Mental State Exam; Nd, Not done; Unk, Unknown.

^a^ MMSE carried out.

## Results

### Pedigrees and Genetic Analysis

**FIG. 1:**
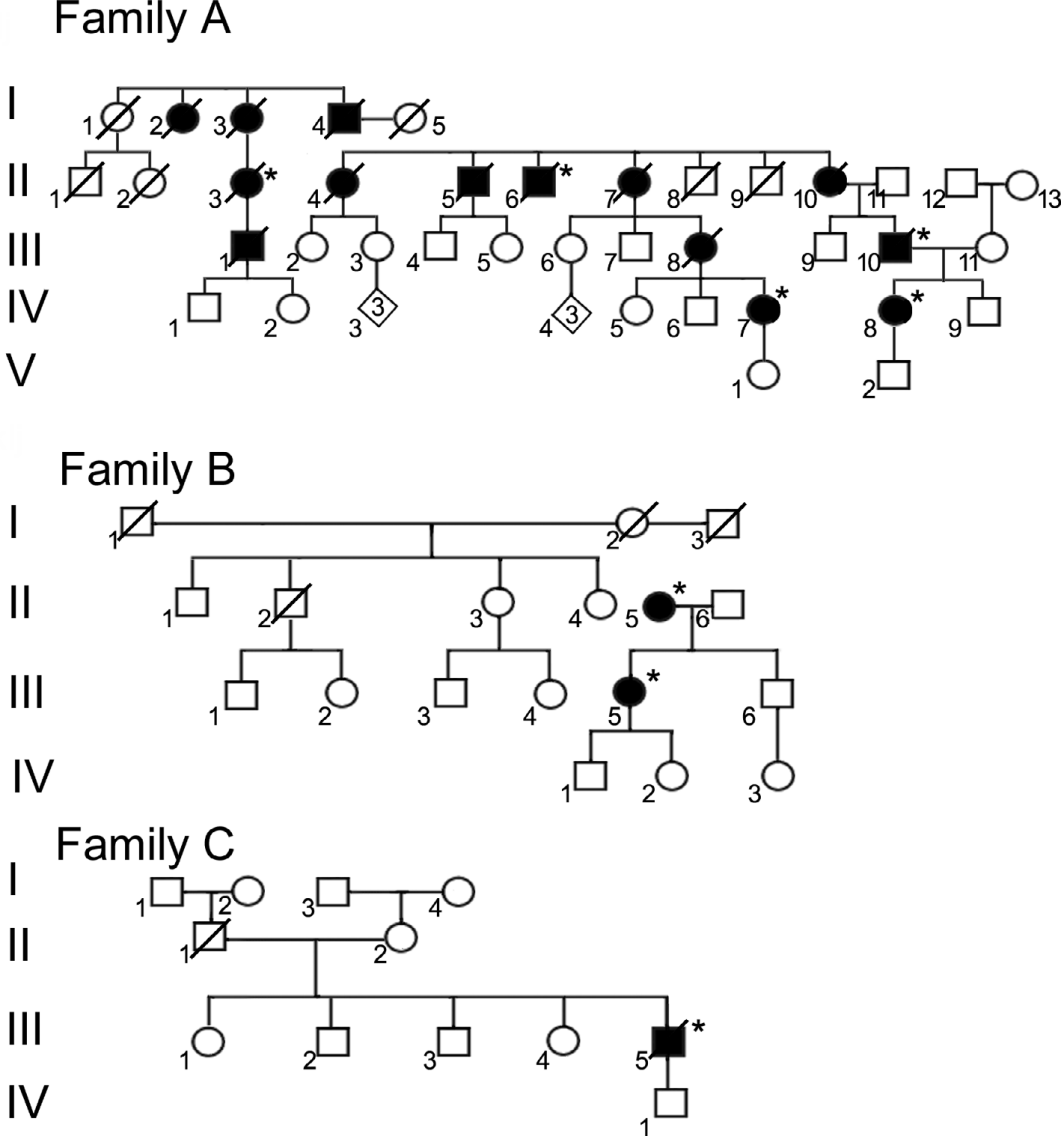
Family trees of affected families; *indicates patients reviewed for this study.

Median age at onset (AAO) was 38 years (Table 1). The median repeat length for the study participants was 52 (range, 45–63) (Table 1). Repeat length was inversely correlated with AAO (correlation coefficient of −0.8144). Of note, A‐IV‐7 had the largest repeat length (63) reported, and manifested with symptoms at the age of 18.^4^ A‐IV‐8 and A‐II‐3, who were 19 and 25 years old, respectively at presentation, both had repeat lengths of 59. The median repeat length of 52 was significantly greater that that derived from a previously reported group of patients from Northern South Africa, predominantly of black Southern African origin (median repeat length, 46, *P* = 0.008).^2^ Patients II‐3, II‐6, and III‐10 from Family A (Fig. 1) were reported in 2007,^5^ and patient A‐IV‐7 formed part of a study comparing HD and HDL2.^2^

### Cognitive and Behavioral Findings

Symptoms at disease onset included both cognitive and motor impairments, with cognitive deficits generally being more severe. Family members were less aware of motor impairment than cognitive symptoms. All but one participant underwent cognitive assessment at some point in their disease course (Table 1). MoCA scores reflected impairments across all domains, although tests for orientation and memory were less severely affected. Cognitive impairment was at times challenging to quantify because of its severity, and because of limited schooling.

Neuropsychiatric symptoms were common in all, evident as behavioral change, memory impairment, depression, anxiety/agitation, irritability, and/or hallucinations. Behavior was challenging to assess because participants were apathetic and often unable to engage in conversation. Participants were typically unable to work or to manage their own affairs. Some were able to groom and feed themselves, and care could still be provided at home for most. However, only one participant was able to perform household chores and supervise children. Visual‐, and to a lesser extent, auditory‐ hallucinations were present in the third‐generation members of pedigree A and B, and in the affected member from family C. Paranoid delusions were also present, and patients were prone to wandering. At the time of the study and 10 years into their illness, participants A‐IV‐7 and A‐IV‐8 were found to have compulsive behavior. A‐II‐3 and A‐III‐10 became aggressive over time; participant A‐III‐10 had a premorbid diagnosis of depression and psychosis. Data on participant A‐II‐3 is very limited, however, disinhibition, aggression, and echolalia were noted.

### UHDRS

Participants A‐II‐3, A‐II‐6, A‐III‐10, and C‐III‐5 only had certain motor domains of the UHDRS assessed based on the availability of their medical records and available video material. On the UHDRS, total scores for dystonia, rigidity, and bradykinesia were all greater than the total score for chorea, with moderate generalized dystonia present in all but two participants. Chorea was absent in two patients; in five, chorea consisted only of mild, small amplitude movements of either the toes, arms, trunk and/or buccal‐oral‐lingual areas (range 1–4 on the UHDRS). A single patient had marked chorea, predominantly involving the arms and face (C‐III‐5). Highest UHDRS scores were in the domains of eye movement abnormalities and bradykinesia (six participants had eye movement evaluation, and all but one scored the highest for this component of the UHDRS). With respect to motor deficits, bradykinesia and tremor were both symptoms and signs in four patients at disease onset. At time of examination, bradykinesia was present in seven patients and tremor was present in six patients.

Specific study findings were as follows, listed according to age at presentation:

A‐IV‐7: presented at age 18 with cognitive slowing, change in behavior, memory impairment, and dysarthria.

A‐IV‐8: presented at age 19 with excessive sleepiness, obsessional behavior, dysarthria, and bilateral upper limb tremor.

A‐II‐3: presented with chorea at age 25 years, and was noted to be demented, although formal cognitive testing was not performed.

A‐II‐6: presented at age 38 years with tremor and cognitive impairment.

B‐III‐5: presented at 38 years with memory impairment, cognitive slowing (poor attention and forgetfulness), progressive deterioration of gait, and psychosis (auditory and visual hallucinations).

A‐III‐10: developed tremor at age 42 years, followed by personality change, and was found to have bradykinesia, tremor, myoclonus, and dementia 4 years later.

C‐III‐5: presented at age 43 years with depression, bradykinesia, rigidity, and upper limb tremor.

B‐II‐5: presented at age 48 years with anxiety and depression, with change in gait and frequent falls and chorea.

### Brain Imaging

Seven of the eight participants had brain magnetic resonance imaging performed. Caudate atrophy and generalized cerebral atrophy were present in all participants. In addition, individuals A‐III‐10, A‐IV‐8, B‐III‐5, and B‐II‐5 showed leukoaraiosis.

## Discussion

HDL2 is increasingly recognized as having a range of clinical presentations.^2^, ^3^, ^5^, ^6^ We report a predominantly cognitive and behavioral presentation, with less pronounced, or even absent, chorea. Albeit speculative, this may represent the effect of large repeat sizes.

The notable clinical findings of this study were that affected individuals presented with cognitive and behavioral symptoms at onset, and in those with concurrent motor symptoms, the cognitive and behavioral features were more significant. Scores from the MMSE and MOCA ranged from 1 to 23, with all but a single participant having a score <15. Patients displayed impaired executive function, with impairment in psychomotor speed.^7^ Early psychiatric symptoms ranged from depression to frank psychosis, and behavioral change was common. Chorea was infrequent and mild. Gait and speech abnormalities were insignificant. Eye movement abnormalities were common, confirming a recent report from South Africa.^2^

Repeat length was inversely correlated with AAO (correlation coefficient of −0.8144), and this study consequently adds to existing evidence that there is a negative correlation between AAO and repeat length in HDL2.^3^ Moreover, we speculate that higher repeat lengths may result in atypical clinical presentations and greater severity of disease, as has been reported in HD.^8^ The population studied included an individual with, to our knowledge, the largest ever reported repeat length (14/63).^4^ In addition, the median repeat length (52) of the study population is significantly larger than the median of 46, which has previously been recorded from a group of patients from Northern Southern Africa who had typical features of HDL2.^2^ The average AAO in this study was 34 years, considerably lower than the 41 years recently reported as being the typical age at onset of motor symptoms.^4^ Furthermore, we report two unique cases of juvenile onset HDL2 (ages 18 and 19), noting that an AAO younger than 20 years has not been previously documented.

The participants in this study had dystonia and rigidity as their predominant motor features, noting that presentations of HDL2 with parkinsonism have been reported from Brazil, the West Indies, and the United States.^9^, ^10^, ^11^ The Westphal variant of HD (juvenile HD), may present with dementia and parkinsonism,^11^, ^13^ and the two cases of juvenile HDL2 reported in our study presented similarly with dementia, dystonia, and rigidity, associated with large repeat expansions (14/63 and 14/59).

It is established in HD that individuals with larger repeat lengths manifest symptoms at an earlier age, and those with 60 or more CAG repeats invariably manifest at age 20 or younger.^12^ The pathogenesis of HD is complex, and it is likely that CAG repeat length is the main driver of disease onset and progression. Somatic expansions because of genetic modifiers may increase the CAG repeat length, and both the rate of expansion, as well as the threshold repeat length, is implicated in disease onset and progression.^13^, ^14^ In addition, genetic modifiers have been identified that influence AAO in HD,^15^ and these may have contributed to variable clinical presentations in the South African patients studied. Haplotype studies have found multiple origins for the HD mutation in ethnically distinct subpopulations of South Africa.^16^ In conclusion, it is important for clinicians to be aware that individuals with HDL2 may present with severe and rapidly progressive cognitive and behavioral impairments, with a variable degree of motor deficit.

## Author Roles

(1) Research Project: A. Conception, B. Organization, C. Execution; (2) Statistical Analysis: A. Design, B. Execution, C. Review and Critique; (3) Manuscript: A. Writing of the First Draft, B. Review and Critique.

H.N.J.: 1B, 1C, 2A, 2B, 2C, 3A

M.G.: 1A, 1C

L.v.H.: 1A, 1B, 1C

R.v.C.: 1A, 1C

A.K.: 1A, 1C, 3B

J.C.: 1A, 1B, 1C, 3A, 3B

## Disclosures

**Ethical Compliance Statement**: This study was granted ethics approval from the Health Research Ethics Committee (HREC) of the University of Stellenbosch (S20/09/258). All participants provided informed consent.

The authors confirm that they have read the Journal's position on issues involved in ethical publication and affirm that this work is consistent with those guidelines.

**Funding Sources and Conflicts of Interest**: No specific funding was received for this work; the authors declare that there are no conflicts of interest relevant to this work.

**Financial Disclosures for Previous 12 Months**: M.G. is a consultant to CHDI Foundation, DSMB Board Chair for AskBio and SOM Therapeutics. J.C. has received grants from The Michael J. Fox Foundation.
